# Efficacy of Retreatment with Oxaliplatin-Based Regimens in Metastatic Colorectal Cancer Patients: The RETROX-CRC Retrospective Study

**DOI:** 10.3390/cancers14051197

**Published:** 2022-02-25

**Authors:** Alessio Amatu, Gianluca Mauri, Federica Tosi, Katia Bencardino, Erica Bonazzina, Viviana Gori, Lorenzo Ruggieri, Sabrina Arena, Alberto Bardelli, Silvia Marsoni, Salvatore Siena, Andrea Sartore-Bianchi

**Affiliations:** 1 Department of Hematology, Oncology, and Molecular Medicine, Grande Ospedale Metropolitano Niguarda, 20162 Milan, Italy; alessio.amatu@ospedaleniguarda.it (A.A.); gianluca.mauri@unimi.it (G.M.); federica.tosi@ospedaleniguarda.it (F.T.); katiabruna.bencardino@ospedaleniguarda.it (K.B.); ericafrancesca.bonazzina@ospedaleniguarda.it (E.B.); viviana.gori@ospedaleniguarda.it (V.G.); lorenzo.ruggieri@ospedaleniguarda.it (L.R.); salvatore.siena@unimi.it (S.S.); 2 Department of Oncology and Hemato-Oncology, Università degli Studi di Milano, 20122 Milan, Italy; 3 IFOM-FIRC Institute of Molecular Oncology, 20139 Milan, Italy; silvia.marsoni@ifom.eu; 4 Candiolo Cancer Institute, Fondazione del Piemonte per l’Oncologia (FPO)-Istituto di Ricovero e Cura a Carattere Scientifico (IRCCS), 10060 Candiolo, Italy; sabrina.arena@unito.it (S.A.); alberto.bardelli@unito.it (A.B.); 5 Department of Oncology, University of Torino, 10060 Candiolo, Italy

**Keywords:** oxaliplatin, rechallenge, reintroduction, continuum-of-care

## Abstract

**Simple Summary:**

The efficacy and safety of oxaliplatin-based regimens in late-care settings have been poorly reported. In 119 mCRC patients, the oxaliplatin retreatment response rate (RR) was 21.6%. The median progression-free survival was 5.1 months. A total of 34/119 (28.6%) discontinued treatments were due to toxicities. Oxaliplatin retreatment produced further RR, but one-third of patients discontinued treatment due to adverse events. Thus, translational studies that improve patient selections are warranted.

**Abstract:**

Background: oxaliplatin with fluoropyrimidine is a “mainstay” regarding the upfront treatment of metastatic colorectal cancer (mCRC). In contrast, the efficacy and safety of oxaliplatin-based regimens in late-care settings have been poorly reported. Methods: we identified a real-world mCRC patient cohort who were re-treated with oxaliplatin, and in which clinicopathological features were retrospectively analyzed to identify efficacy–predictive determinants (RETROX-CRC study). Results: of 2606 patients, 119 fulfilled the eligibility criteria. Oxaliplatin retreatment response rate (RR) and disease control rate (DCR) were 21.6% (CI 14.4–31.0%), and 57.8% (CI 47.7–67.4). A trend towards better RR and DCR was observed among patients who had first oxaliplatin in an adjuvant setting; a poorer outcome was observed if two or more intervening treatments were delivered. Median progression-free survival (PFS) was 5.1 months (95%CI 4.3–6.1), reducing to 4.0 months (95%CI 3.07–5.13) if oxaliplatin was readministered beyond third-line (HR 2.02; 1.25–3.25; *p* = 0.004). Safety data were retrieved in 65 patients (54.6%); 18.5% (12/65) and 7.7% (5/65) had G3–4 toxicities. Toxicities led to discontinuation in 34/119 (28.6%). Conclusions: oxaliplatin retreatment produced further RR in around one-fifth of patients and DCR 57.8%. Efficacy decreased in more pre-treated patients and around one-third of patients discontinued treatment due to adverse events. Translational studies improving patient selection are warranted.

## 1. Introduction

Half of patients diagnosed with colorectal cancer (CRC) present or will develop distant metastases [1], translating into a median overall survival (OS) of 2.5 years [2]. The evolution of systemic therapies in metastatic patients (mCRC) has not been transformative as in other solid tumors, mostly due to the relatively limited number of patients that could be successfully treated with either targeted therapy or immunotherapy under the paradigm of precision medicine [3,4,5,6,7,8]. Thus, chemotherapy remains as the “workhorse” of treatment in mCRC. Notably, all front-line regimens use 5-fluorouracil (5-FU), supplemented by folinic acid, as a backbone to which either oxaliplatin (a DNA cross-linker) or irinotecan (a topoisomerase-1 inhibitor) are added. Upon progression, the two regimens are switched as second-line. Albeit equivalent in efficacy, oxaliplatin-based regimens are used more than irinotecan-based ones [3,9]. Oxaliplatin, in combination with fluoropyrimidines, reaches a response rate (RR) around 60% in the first-line setting [10,11,12]. Based on these data, oxaliplatin plus 5-FU, or its oral prodrug capecitabine (FOLFOX and CAPOX regimens, respectively), are the “mainstreams” of both adjuvant and advanced treatments [3,4,13]. However, oxaliplatin, which is weighed by potentially severe and persistent side effects such as persistent peripheral sensory neuropathy, might greatly affects patients quality of life [14,15,16,17]. 

Considering oxaliplatin efficacy and its low cost, oxaliplatin is also readministered in pre-treated patients in clinical practice, particularly if there are no clinical trials available [18]. However, clinical evidence supporting this strategy is conflictual and based on small patient cohorts, making oxaliplatin retreatment not currently recommended by mCRC clinical guidelines [3,4]. In this regard, we recently conducted an extensive critical review of the available literature, allowing the identification of eight full-text articles and four abstracts discussing oxaliplatin retreatment in mCRC, which found a RR of oxaliplatin readministered together with 5-FU ranging between 6 and 31%, a disease control rate (DCR) between 39 and 79%, and median progression-free survival (PFS) from 3 to 7 months [18]. Nevertheless, there is still no consensus on if or when oxaliplatin retreatment should be integrated in the continuum of care of mCRC patients [18]. Safety wise, grade 3 or higher toxicities are experienced by 15–20% of the mCRC oxaliplatin-re-treated population. Hematological adverse events and acute allergic reactions are the most predominant [18]. Interestingly patient selection criteria and retreatment strategies were widely heterogenous among different studies, hampering the assessment of the real impact of oxaliplatin retreatment in mCRC patients [18]. Finally, many trials and reports have included the addition of biological agents, such as anti-VEGF or anti-EGFR, to the oxaliplatin retreatment backbone, further complicating the interpretation of the efficacy results [19,20,21]. In addition, a consensus on validated clinical criteria selecting mCRC patients for oxaliplatin retreatment is lacking [18,22]. Thus, several issues remain to be addressed to optimize the opportunity of retreating mCRC patients with oxaliplatin-based regimens. 

In the present RETROX-CRC study, we present the largest available retrospective cohort of mCRC patients re-treated with oxaliplatin, along with data about the effectiveness and safety of such a strategy. The purpose of this study is to assess the outcome to oxaliplatin retreatment regimens and to explore clinical criteria to better select patients.

## 2. Materials and Methods

### 2.1. Patient’ Selection and Inclusion Criteria 

In this cohort study, referred to as RETROX-CRC, conducted at Niguarda Cancer Center, Milan, Italy, we retrospectively reviewed the available clinical charts of mCRC patients treated at our institution from 1 March 2002 to 29 May 2019. We selected patients who were treated at least twice with oxaliplatin-based regimens throughout their course of care, with evidence of progression of disease in between the two oxaliplatin-based regimens. Given that a consensus for defining oxaliplatin sensitivity in CRC is still lacking, response and time to progression after the first oxaliplatin regimen were considered as variables to be analyzed rather than inclusion criteria. Patient selection and retrospective charts reviewing process were performed by four authors (G.M., E.B., V.G., and L.R.). Clinicopathological features, treatment outcomes, and safety data were retrospectively collected according to electronic clinical records. Efficacy endpoints and adverse events were assessed based on patient chart reports, according to the Response Evaluation Criteria in Solid Tumors (RECIST) criteria and the Common Terminology Criteria for Adverse Events (CTCAE). Furthermore, we collected molecular data, including *RAS*, *BRAF*, and *ERBB2* genes, and the mismatch repair (MMR) protein status, according to what was reported in the clinical charts (Supplementary Table S1). In particular, in the majority of cases (58.8%, 70/119), patients were tested for *RAS* and *BRAF* status at the Pathology Department of Niguarda Cancer Center using high resolution melting (HRM), Sanger sequencing, and allele specific polymerase chain reaction (AS-PCR). MMR status was assessed using PCR as recommended by the European Society of Medical Oncology (ESMO) guidelines [3]. HER2 status was assessed by immunohistochemistry (IHC) and fluorescence in-situ hybridization (FISH), following CRC specific criteria published elsewhere [23]. Moreover, next generation sequencing (NGS) was performed in selected patients (Foundation Medicine assay).

### 2.2. Sample Size and Statistical Analysis

No formal sample size calculation was conducted due to the retrospective nature of our study. However, before the analysis, we estimated that with a sample size of at least 100 patients, we had 80% power (1-β) and a 2.5% type I error (α), to observe a clinically significant improvement of at least 5% RR when the true RR was at least 15% (H_1_), considering that clinical trials after the second-line showed less than a 5% response rate (H_0_) with the available last-line treatment options (regorafenib or trifluridine–tipiracil). Other outcomes collected included PFS and DCR after oxaliplatin reintroduction, along with clinical variables (Supplementary Table S1); clinical and molecular variables with less than 15% missing cases were included in the statistical analysis. Exploratory analyses included univariate and multivariate logistic regression of OR for RR and DCR. We explored (with a Cox regression model) the correlations of the main clinical variables with PFS. All reported confidence intervals (CI) are 95% unless otherwise specified. All analyses were conducted with R statistical software [24,25].

## 3. Results

### 3.1. Clinicopathological Features and Outcomes to the First Oxaliplatin Administration

Among a total of 2606 mCRC patients reviewed, 119 were re-treated with an oxaliplatin-based regimen (4.6%) and were included in the present study. The main clinicopathological features are summarized in Supplementary Table S1. A total of 56.3% patients were men; the median age at diagnosis of the entire cohort was 56.91 [23.98, 79.08] and 30.3% were early-onset CRC (EO-CRC) diagnosed at an age younger than 50 [26], which was possibly “enriched” because of the attitudes in performing multiple therapeutic lines in young individuals [27]. Most of the patients were diagnosed in the stage IV disease, left-sided, or rectal primary tumor, and had their primary tumors resected. The median number of systemic medical treatments was 2 (range 0–7). *RAS* mutation prevalence, considering standard molecular assessment, was similar to the general mCRC population (54/115, 46.9%) [28]. Our cohort was also quite enriched in the *BRAF^V600E^* mutant (13/95, 13.7%) [29] and more in *ERBB2*-amplified mCRC (11/80, 13.8%) if compared to the general population, likely due to referrals of such subsets of patients at our center for clinical trials [30,31,32]. Half of our cohort received the first oxaliplatin-based regimen as an adjuvant treatment and most were treated with the FOLFOX regimen (Supplementary Table S1). Interestingly, median time-to-progression (mTTP) was similar in patients receiving the first oxaliplatin-based regimen in an adjuvant or metastatic setting: 15.3 months and 15.2 months, respectively. Among patients who received the first oxaliplatin-based regimens in a metastatic setting, 62.7% (37/59) of patients received at least “a third drug, other than 5-FU, concomitantly to the first oxaliplatin administration, and 88.1% (52/59) had (at least) disease control as the best response (Supplementary Table S1). The median first oxaliplatin-based treatment duration was 6.0 months, both in the adjuvant and metastatic settings. In the whole cohort, 9.2% (11/119) patients progressed while receiving the first oxaliplatin-based regimen. Among the remaining, 48.7% (58/119) discontinued the first oxaliplatin-based regimen at the end of the 6 months of standard adjuvant courses, 24.4% (29/119) after 6 months of treatment in the metastatic setting to switch to a maintenance oxaliplatin-free regimen or to undergo a therapeutic holiday, while the remaining 17.7% (21/119) discontinued to undergo a liver resection for metastatic disease. A considerable portion of patients in our cohort was heavily pre-treated, with 29.4% of patients receiving three or more intervening treatments, including surgical procedures and/or local approaches such as radiotherapy, before oxaliplatin retreatment. Only 12.6% of patients received regorafenib and/or trifluridine–tipiracil since most of them were treated before approval of these two drugs in Italy (Supplementary Table S1).

### 3.2. Efficacy of Oxaliplatin Retreatment

According to the definitions of the context of oxaliplatin retreatment that we previously provided elsewhere [18], 9.2% of patients from our cohort received a rechallenge and 90.8% an oxaliplatin reintroduction. Median oxaliplatin retreatment duration was 2.8 months. Overall, 85.7% (102/119) of mCRC patients were evaluable for response to oxaliplatin retreatment while 14.3% were not evaluable due to toxicities leading to treatment interruption (see paragraph “Safety and Tolerability”).

**Figure 1:**
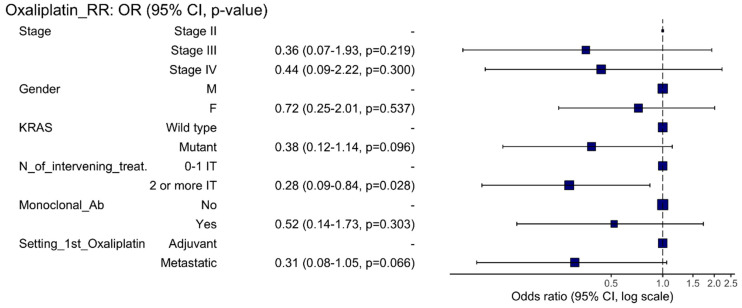
Forest plot depicting the hazard ratios of the multivariate analysis of oxaliplatin retreatment response rate (RR). Keys: M = male; F = female; N = number; Ab = antibody; IT = intervening treatment.

The RR of all evaluable patients was 21.6% (CI 14.3–31.0; *p*-value < 0.001). RR among those who received the first oxaliplatin-based regimen in the adjuvant and metastatic settings were 28.3% (CI 17.2–42.6) and 14.3% (CI 6.4–27.9), respectively. Main clinical variables were not significantly associated with RR (Supplementary Table S2). The odds ratio for response was analyzed in a univariate and multivariate logistic model (Table 1). At the multivariate analysis, a higher number of intervening treatments was significantly associated with a worse outcome to oxaliplatin retreatment (Figure 1). Contrarily, a trend towards a better RR was present among patients receiving the first oxaliplatin-based regimen in the adjuvant setting (Table 1 and Figure 1). We observed 57.8% DCR (95%CI 47.7–67.4) in the 102 evaluable patients (Supplementary Table S3). Both the logistic regression and the multivariate analysis showed a worse DCR in those patients receiving more than two intervening treatments before oxaliplatin re-administration and if oxaliplatin retreatment was pursued beyond the second-line of treatment (Supplementary Table S4 and Supplementary Figure S1). 

**Figure 2:**
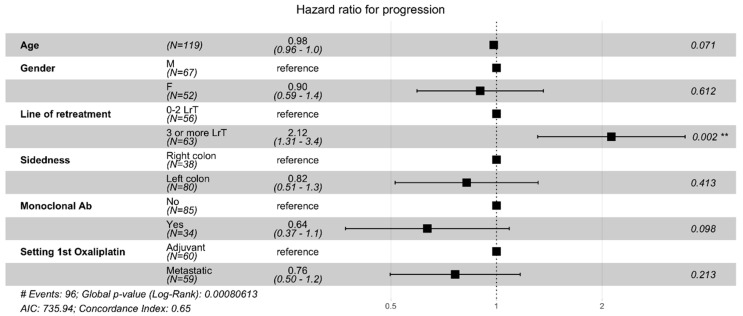
Forest plot depicting the hazard ratios of the multivariate analysis of oxaliplatin retreatment progression-free survival (PFS). Keys: M = male; F = female; LrT = line of retreatment; ** = statistically significant.

Data on PFS were available in all patients enrolled in our cohort. Among the entire cohort of oxaliplatin re-treated patients, median PFS was 5.1 months (95%CI 4.3–6.1), reducing to 4.03 months (95%CI 3.07–5.13) when oxaliplatin was reintroduced in the third or later line of treatment. The Cox regression model showed that the addition of anti-EGFR or anti-VEGF reduced the risk of progression at univariate analysis (HR 0.50, CI 0.31–0.81; *p* = 0.005), although the effect was not confirmed in the multivariate model (HR 0.68; CI 0.40–1.15; *p* = 0.147). The risk of progression if retreating patients with oxaliplatin in the third-line or beyond was more than doubled when compared to the first- or second-lines, both at univariate and multivariate analyses (HR 2.02; CI 1.25–3.25; *p* = 0.004) (Table 2 and Figure 2). 

### 3.3. Safety and Tolerability 

At the time of oxaliplatin retreatment, data on residual neuropathy due to first oxaliplatin administration were retrieved in 69/119 (58.0%) patients. Among these patients, 9/69 (13.0%) had pre-existing neuropathy, of which, 6/9 (66.7%) had G1, 1/9 (11.1%) G2, 1/9 (11.1%) G3, and 1/9 (11.1%) an unknown grade. 

Oxaliplatin retreatment toxicity data were available in 65/119 (54.6%) patients, of whom, 18.5% and 7.7% had G3 and G4 toxicities, respectively. Table 3 describes all toxicities experienced by patients enrolled in our cohort who underwent oxaliplatin retreatment. The most common side effects were fatigue, nausea, hematological (neutropenia or thrombocytopenia), and peripheral sensory neuropathy. In particular, the latter was identified in 20/65 (30.7%) patients; of whom, 60.0% had G1, 25.0% G2, and 5% G3 neuropathy (Table 3). Among the entire cohort, 34/119 (28.6%) patients experienced an adverse event leading to treatment discontinuation, and in 20/34 (58.8%) cases, a hypersensitivity acute reaction was the cause of treatment discontinuation. A total of 4/34 (11.8%) patients were diagnosed with oxaliplatin-immune induced syndrome (OIIS) [16,17]. 

In order to evaluate potential molecular mechanisms of sensitivity to re-administration of oxaliplatin, NGS analysis (Foundation Medicine assay) results were reviewed and available in 6/22 (27.3%) patients, who achieved at least disease control (PR + SD) (27.3%). Among these, 4/6 (66.7%) had at least one mutation in genes engaged in the DNA damage response (DDR) pathways, such as *BRIP1* (*FANCJ*), *ATRX*, *FANCA*, and *CHEK2* (Supplementary Figure S2) [33,34].

## 4. Discussion

Data on oxaliplatin efficacy in the late-line spaces of CRC therapy are conflicting while the acute and long-term safety issues of this DNA alkylator are well known [14,18,35]. This fact, combined with the heterogeneity of clinical criteria used in prior reports for patient selection [18,21], and the lack of predictive biomarkers, explains the absence of oxaliplatin retreatment as a therapeutic option in both NCCN and ESMO CRC guidelines [2,3,4]. Not surprisingly, despite scarce and heterogeneous supporting evidence, oxaliplatin-based regimes are used beyond second-line in mCRC, as we confirm in our study, the largest available [18,22], in which around 5% of patients received oxaliplatin retreatment out of 2606 cases referred to Niguarda Cancer Center over a period of 17 years.

On the other hand, our real-world study indicates that a potential for oxaliplatin retreatment might have been overlooked. In the continuum of care of mCRC patients, regorafenib, a multikinase inhibitor, and trifluridine–tipiracil (TAS-102), a fluoropyrimidine with improved bioavailability, are the only two guideline-approved drugs for late treatment [3,4,36,37]. A recent systematic review that evaluated the studies on various treatment strategies for late line mCRC with the aim of identifying an optimal approach, indeed suggested that TAS-102 or regorafenib should be used before any rechallenge beyond the second-line in mCRC [38]. However, this review was unable to conclude on oxaliplatin reintroduction or rechallenges due to lack of appropriate trials [38]. Instead, it confirmed the results of approval trials with regorafenib and TAS-102, which share similar efficacy data with RRs, around 5%, and a median PFS ranging from 2 to 5 months, when compared to placebo [36,37]. The RR to oxaliplatin-based regimens in RETROX was slightly above 20% and increased to almost 30% among patients who received their first oxaliplatin-based regimens as adjuvant treatments, and the median PFS was around 5 months. While non-randomized and historical comparisons, especially of time-dependent outcomes, provide the lowest and worst levels of evidence in medicine, our results are supported by a recent study, small, and (so far) published only in abstract format, which compared a retrospectively determined single-institution response, PFS, and survival data of patients re-treated with FOLFOX or treated with regorafenib [39]. This study showed a significant advantage for FOLFOX in all three efficacy outcomes and specifically reported a 3% RR for regorafenib in contrast to a FOLFOX RR of 25%, thus superimposable to the RR we found in RETROX. 

Accurate patient selection improves the therapeutic index of any regimen, including oxaliplatin retreatment in mCRC. Indeed, even if oxaliplatin retreatment has been reported as a potential treatment option, particularly in mCRC patients with at least 6 months of disease-free or progression-free survival with prior oxaliplatin, neurotoxicity and hypersensitivity reactions should be carefully monitored [22]. While we were unable to identify a specific predictive clinical marker, our cohort included mainly patients who did not progress while on the first oxaliplatin treatment (reintroduction setting). Could that justify a response rate of 20%? The response and response duration to carboplatin are well established criteria for deciding platinum-based retreatment in ovarian cancer [40]; thus, it is not surprising that response to oxaliplatin retreatment seems to follow the same pattern in CRC. In ovarian cancer, the most important predictor of response to carboplatin retreatment is the length of PFS between consecutive treatment sessions [40,41]. In CRC, the closest information on the potential role of the oxaliplatin-free interval as a predictive marker comes from the FOLFOX stop-and-go approach adopted in the OPTIMOX1 and 2 trials originally designed to mitigate oxaliplatin neurotoxicity [42]. In a pooled analysis of the two trials following FOLFOX reintroduction (as second-line) both RR and PFS almost doubled when the oxaliplatin-free interval was ≥6 months (22% vs. 14% and 5.5. vs. 3.0 months in patients, respectively, for oxaliplatin-free interval < or >6 months) [42]. Of note, the equivalent to second line oxaliplatin reintroduction attempted in the OPTIMOX trials was considered unsuccessful by the authors, not for lack of activity but rather because of feasibility issue (too many protocol violations) [42]. We were unable to reliably reconstruct the oxaliplatin-free interval in the RETROX patients. However, using as a proxy the number of intervening treatments delivered between the first oxaliplatin regimen and that at retreatment, we found an inverse correlation, in terms of RR and PFS. Conversely, a trend towards a positive correlation was found when the first oxaliplatin regimen was delivered as an adjuvant therapy. These findings advocate offering oxaliplatin retreatment to patients who do not progress while receiving their first oxaliplatin-based regimens and not beyond the third-line setting. 

Predictive biomarkers of response for the drugs currently used in the late treatment space are a truly unmet clinical need in mCRC, especially due to their associations with severe and often persisting side effects [14,16,18,35]. Indeed, beyond clinical selection criteria, the identification of molecular biomarkers might provide further criteria to improve patient selection towards oxaliplatin retreatment. In this regard, we recently found that oxaliplatin efficacy is linked to alterations in the DNA damage response (DDR) and homologous recombination (HR) machinery [33,34]. An in vitro screen of a large number of human CRC cell lines, revealed pharmacologic cross-sensitivity between oxaliplatin and the PARP inhibitor olaparib, which was confirmed and exploited with therapeutic success in a preclinical trial combining PARPi and oxaliplatin in mCRC patient-derived-xenografts [33]. We also reported that, when the tumors of a small subset of RETROX patients were genomically profiled with a customized next generation sequencing (NGS) panel, many harbored selected DDR mutations [33,34]. We were unable to establish a specific pattern between DDR alterations and response to oxaliplatin; however, this might be because the whole spectrum of DDR deregulation in mCRC is not fully captured by the sole genomic analysis, and the numerous variants of unknown significance (VUS), and other means to identify patients affected by CRC harboring DDR deficiencies, are still under evaluation [33,34]. Further translational studies are warranted to verify if DDR alterations might act as molecular biomarkers for oxaliplatin sensitivity in CRC patients. 

Our study has several limitations, as do all retrospective studies based on real-world data. The first is represented by its retrospective nature, hampering the full availability of molecular and overall survival data from enrolled patients. The second is the potential for selection bias. As an example, we might have underestimated the prevalence of oxaliplatin retreatment in late mCRC because data were derived from a comprehensive cancer center entering a larger than average proportion of referred mCRC patients into clinical trials. We might also have underestimated safety issues. We detected severe toxicities (G3–4) in approximately one fourth of the patients, which is on the lower side of the range of toxicity for oxaliplatin, but we were able to retrieve reliable toxicity data in slightly more than half of the treated cohort. Thirdly, we were unable to perform a comprehensive comparison between reintroduction and rechallenge strategies because the paucity of rechallenged cases (9/119), which would have impaired the statistical soundness of the analysis. Oxaliplatin retreatment was tested in a small randomized phase II trial and two non-randomized real world trials, which all reported impressive median OS, but the design of the first study also permitted oxaliplatin reintroduction in patients who had not progressed in their earlier regimens, and the results of the latter two must be interpreted with caution due to the small sample sizes and absence of control groups [43,44,45].

## 5. Conclusions

In conclusion, with the limitations intrinsic to the retrospective nature of the study, RETROX has shown that oxaliplatin retreatment could be safely delivered to real-world patients in late care settings and provide further objective response rates in around one-fifth of patients. Further translational studies, to identify biomarkers linking the tumor biology to oxaliplatin sensitivity, are warranted and ongoing. In the meanwhile, while visibly, only a prospective randomized trial would allow drawing truly unbiased conclusions, RETROX results indicate that oxaliplatin might represent a “salvage” option, especially for patients who firstly receive the drug as an adjuvant treatment or progress off-therapy after their first oxaliplatin-based treatment.

## Figures and Tables

**Table 1:** Odds ratios at univariate and multivariate analyses of metastatic colorectal cancer patients who were evaluable for response rate (RR) with oxaliplatin retreatment. Keys: SD = stable disease; PR = partial response; CR = complete response; PD = progressive disease; St.Dev = standard deviation; OR = odds ratio. * = statistically significant.

Characteristic	Variables	SD + PD	PR + CR	OR (Univariable)	OR (Multivariable)
Primary tumor location	Right colon	24 (77.4)	7 (22.6)	-	-
	Left colon and rectal	56 (78.9)	15 (21.1)	0.92 (0.34–2.66, *p* = 0.870)	-
Stage at initial diagnosis	Stage II	7 (63.6)	4 (36.4)	-	-
	Stage III	31 (77.5)	9 (22.5)	0.51 (0.12–2.29, *p* = 0.355)	0.36 (0.07–1.93, *p* = 0.219)
	Stage IV	42 (82.4)	9 (17.6)	0.37 (0.09–1.67, *p* = 0.177)	0.44 (0.09–2.22, *p* = 0.300)
Age at initial diagnosis	Mean (St.Dev.)	55.3 (11.6)	57.6 (10.6)	1.02 (0.98–1.06, *p* = 0.406)	-
Gender	Male	44 (75.9)	14 (24.1)	-	-
	Female	36 (81.8)	8 (18.2)	0.70 (0.25–1.82, *p* = 0.470)	0.72 (0.25–2.01, *p* = 0.537)
*KRAS* status	Wild type	40 (72.7)	15 (27.3)	-	-
	Mutant	37 (84.1)	7 (15.9)	0.50 (0.18–1.34, *p* = 0.181)	0.38 (0.12–1.14, *p* = 0.096)
Number of intervening treatments *	0–1	34 (70.8)	14 (29.2)	-	-
	≥2	46 (85.2)	8 (14.8)	0.42 (0.15–1.10, *p* = 0.083)	0.28 (0.09–0.84, *p* = 0.028)
Third drug concomitant to oxaliplatin retreatment	No	58 (79.5)	15 (20.5)	-	-
	Yes	22 (75.9)	7 (24.1)	1.23 (0.42–3.35, *p* = 0.691)	0.52 (0.14–1.73, *p* = 0.303)
Line of oxaliplatin retreatment	0–2	36 (75.0)	12 (25.0)	-	-
	≥3	44 (81.5)	10 (18.5)	0.68 (0.26–1.76, *p* = 0.428)	-
First oxaliplatin-based regimen setting	Adjuvant	38 (71.7)	15 (28.3)	-	-
	Metastatic	42 (85.7)	7 (14.3)	0.42 (0.15–1.11, *p* = 0.091)	0.31 (0.08–1.05, *p* = 0.066)

**Table 2:** Odds ratios at univariate and multivariate analyses of metastatic colorectal cancer patients who were evaluable for median progression-free survival (mPFS) with oxaliplatin retreatment. * = statistically significant.

Characteristic	Variables	Patients	HR (Univariable)	HR (Multivariable)
Age at initial diagnosis	Mean (SD)	55.9 (11.2)	0.98 (0.96–1.00, *p* = 0.053)	0.98 (0.96–1.00, *p* = 0.077)
Gender	Male	67	-	-
	Female	52	0.97 (0.65–1.46, *p* = 0.884)	0.91 (0.60–1.38, *p* = 0.653)
Primary tumor location	Right colon	38	-	-
	Left colon and rectal	80	0.89 (0.58–1.37, *p* = 0.589)	0.81 (0.51–1.29, *p* = 0.369)
Number of intervening treatments *	0–1	56	-	-
	2 or more	63	1.92 (1.26–2.90, *p* = 0.002)	-
Third drug concomitant to oxaliplatin retreatment	No	85	-	-
	Yes	34	0.50 (0.31–0.81, *p* = 0.005)	0.68 (0.40–1.15, *p* = 0.147)
Line of oxaliplatin retreatment	0–2	56	-	-
	3 or more	63	2.17 (1.43–3.28, *p* < 0.001)	2.02 (1.25–3.25, *p* = 0.004)
First oxaliplatin-based regimen setting	Adjuvant	60	-	-
	Metastatic	59	1.08 (0.72–1.62, *p* = 0.703)	-

**Table 3:** Oxaliplatin retreatment adverse events, according to the Common Terminology Criteria for Adverse Events (CTCAE) retrieved in our retrospective patients’ charts review. Keys: * = both patients who experienced these adverse events were also receiving bevacizumab together with oxaliplatin and 5-fluorouracil; N = absolute number of patients.

Event	On Treatment Toxicities (N = 65/119 (54.6%) Patients)	
	Any N, (%)	Grade ≥ 3 N, (%)
Fatigue	25 (38.5)	4 (6.1%)
Nausea	24 (36.9%)	1 (1.5%)
Peripheral neuropathy	20 (30.7%)	1 (1.5%)
Neutropenia	13 (20.0%)	12 (18.5%)
Diarrhea	12 (18.5%)	0 (0.0%)
Platelet decrease	7 (10.7%)	1 (1.5%)
Mucositis	6 (9.2%)	0(0.0%)
Vomiting	5 (7.7%)	1 (1.5%)
Stypsis	4 (4.6%)	0 (0.0%)
Skin rash	3 (4.6%)	0 (0.0%)
Dysgeusia	3 (4.6%)	0 (0.0%)
Other not specified hematologic toxicities	2 (3.1%)	0 (0.0%)
Anemia	1 (1.5%)	0 (0.0%)
Loss of appetite	1 (1.5%)	0 (0.0%)
Transaminase increase	1 (1.5%)	0 (0.0%)
**Adverse events leading to treatment discontinuation (N = 34/119 (28.6%) patients)**		
Hypersensitivity reactions	20 (58.8%)	
Not specified acute reactions	5 (14.7%)	
OIIS (oxaliplatin immune-induced syndrome)	4 (11.8%)	
Bone marrow toxicities	2 (5.9%)	
Acute neurotoxicity	1 (2.9%)	
Uncontrolled hypertension *	1 (2.9%)	
Acute coronary syndrome *	1 (2.9%)	

## Data Availability

The datasets used and/or analyzed during the current study are available from the corresponding author upon reasonable request.

## Supplementary Materials

The following supporting information can be downloaded at: https://www.mdpi.com/article/10.3390/cancers14051197/s1. Supplementary Figure S1: Forest plot depicting the hazard ratios of the multivariate analysis of oxaliplatin retreatment disease control rate (DCR). Supplementary Figure S2: Prevalence of standard (*RAS*, *BRAF* and *MMR*) and DNA damage response (DDR) alterations in 6 metastatic colorectal cancer (mCRC) patients who underwent next generation sequencing (NGS). Supplementary Table S1: Full list of clinicopathological features, outcome to first oxaliplatin-based regimens and to oxaliplatin retreatment regarding the entire cohort of 119 metastatic colorectal cancer patients re-treated with oxaliplatin-based regimens throughout their course of disease. Supplementary Table S2: Logistic regression analysis of response rate (RR) to oxaliplatin retreatment. Supplementary Table S3: Logistic regression analysis of overall disease control rate (DCR) with oxaliplatin retreatment. Supplementary Table S4: Odds ratios at univariate and multivariate analyses of metastatic colorectal cancer patients who were evaluable for disease control rate (DCR) with oxaliplatin retreatment.
