# Supplementary material for: Efficacy of Retreatment with Oxaliplatin-Based Regimens in Metastatic Colorectal Cancer Patients: The RETROX-CRC Retrospective Study
Source: Cancers (Basel). 2022 Feb 25;14(5):1197. doi: 10.3390/cancers14051197 (PMC8909235; doi:10.3390/cancers14051197)
Supplement: Supplementary file 1 [file cancers-14-01197-s001.zip › cancers-1554466-supplementary.pdf]

Supplementary Table S1. Full list of clinico-pathological features, outcome to first oxaliplatin-based regimens and to oxaliplatin retreatment regarding the entire cohort of 119 metastatic colorectal cancer patients retreated with oxaliplatin-based regimens throughout their course of disease.

**Characteristics**	**Patients (N=119)**
Median age at diagnosis [median (range)] - yr	56.91 [23.98, 79.08]
Age < 50 yr at diagnosis – no. (%)	36 (30.3)
Gender	
Male	67 (56.3)
Female	52 (43.7)
Stage at initial diagnosis – no. (%)	
Stage I	0 (0.0)
Stage II	12 (10.1)
Stage III	47 (39.5)
Stage IV	60 (50.4)
Overall Survival (median [range]) - ms	51.53 [14.77, 175.47]
Primary tumor location – no. (%)	
Right sided	38 (32.0)
Left sided or rectal	80 (67.2)
Missing	1 (0.8)
Primary tumor resected – no. (%)	
Yes	110 (92.4)
No	9 (7.6)
KRAS molecular assessment – no. (%)	
Mutant	52 (43.7)
Wild-type	63 (52.9)
Missing	4 (3.4)
NRAS molecular assessment – no. (%)	
Mutant	2 (1.7)
Wild-type	61 (51.3)
Missing	56 (47.0)
BRAF molecular assessment – no. (%)	
V600E mutant	13 (10.9)
Wild-type	82 (68.9)
Missing	24 (20.2)
MMR molecular assessment – no. (%)	
Microsatellite unstable	2 (1.7)
Microsatellite stable	50 (42.0)
Missing	67 (56.3)
ERBB2 molecular assessment – no. (%)	
Amplified	11 (9.2)
Not amplified	59 (49.6)
Missing	49 (41.2)
First oxaliplatin based regimen – no. (%)	
FOLFOX	66 (55.5)
XELOX	16 (13.4)
FOLFOX + bevacizumab	23 (19.4)
XELOX + bevacizumab	2 (1.7)
FOLFOX + cetuximab	2 (1.7)
FOLFOX + panitumumab	3 (2.5)
XELOX + panitumumab	1 (0.8)
FOLFOXIRI	1 (0.8)
FOLFOXIRI + bevacizumab	4 (3.4)
FOLFOXIRI + cetuximab	1 (0.8)
Setting of first oxaliplatin administration – no. (%)	
Adjuvant	60 (50.4)
Metastatic	59 (49.6)
Best response to first oxaliplatin administration – no. (%)	
Complete response	4 (3.4)
Partial response	32 (26.9)
Stable disease	16 (13.4)

Progressive disease	1 (0.8)
*Not assessable **	66 (55.5)
Patients progressing while receiving first oxaliplatin-based regimen – no. (%)	
Yes	11 (9.2)
No	108 (90.8)
Median time-to-progression (mTTP) after first oxaliplatin-based regimen according to setting of administration – mo. (range)	
*Adjuvant oxaliplatin (N=59**)*	15.3 (4.1-66.2)
Metastatic oxaliplatin (N=59)	15.2 (2.1-34.4)
Treatments administered to patients among the two oxaliplatin-based regimens*** - no. (%)	
0 - 1	56 (47.1)
> 2	63 (52.9)
Patients who received regorafenib or trifluridine-tipiracile (TAS-102) before oxaliplatin retreatment – no. (%)	
None	104 (87.4)
Regorarafenib	8 (6.7)
Trifluridine-tipiracile	1 (0.8)
Both	6 (5.1)
Surgical resection for metastatic disease following first oxaliplatin administration – no. (%)	
Yes	28 (23.5)
No	91 (76.5)
Line of oxaliplatin retreatment – no. (%)	
1 - 2	56 (47.1)
≥ 3	63 (52.9)
Second oxaliplatin based regimen – no. (%)	
FOLFOX	74 (62.2)
FOLFOX + BEVACIZUMAB	24 (20.2)
FOLFOX + CETUXIMAB	1 (0.8)
FOLFOX + PANITUMUMAB	5 (4.2)
FOLFOXIRI + BEVACIZUMAB	2 (1.7)
XELOX	11 (9.2)
XELOX + BEVACIZUMAB	2 (1.7)
PFS_2 (median [range])	4.40 [0.50, 120.63]
Oxaliplatin retreatment response rate (RR) – no. (%)	
Yes	22 (18.5)
No	80 (67.2)
Not assessable	17 (14.3)
Surgery after oxaliplatin retreatment – no. (%)	
Yes	9 (7.6)
No	110 (92.4)
Best response to first oxaliplatin administration – no. (%)	
Complete response	1 (0.8)
Progressive disease	43 (36.1)
Partial response	21 (17.7)
Stable disease	37 (31.1)
Not assessable	17 (14.3)

Keys: *=patients with missing data or who received first oxaliplatin-based regimen in the adjuvant setting; **=in one patient time-to-progression was not available; ***=treatments encompass all treatment approaches (medical, surgical or local such as radiotherapy and others). Legend: yr=years; ms=months; no.=absolute number; PFS_2=progression-free survival with oxaliplatin retreatment.

Supplementary Table S2. Logistic regression analysis of response rate (RR) to oxaliplatin retreatment.

**Characteristic**	**Variables**	**PD+SD (N,%)**	**PR+CR (N,%)**	**Significance (p)**
Oxaliplatin retreatment response rate		80 (78.4)	22 (21.7)	-
Primary tumour location	Right colon	24 (30.0)	7 (31.8)	1.000
	Left colon or rectal	56 (70.0)	15 (68.2)	
Stage at diagnosis	Stage II	7 (8.8)	4 (18.2)	0.385
	Stage III	31 (38.8)	9 (40.9)	
	Stage IV	42 (52.5)	9 (40.9)	
Age at initial diagnosis	Median (IQR)	56.9 (46.5 to 63.3)	59.7 (53.4 to 63.6)	0.345
Gender	Male	44 (55.0)	14 (63.6)	0.630
	Female	36 (45.0)	8 (36.4)	
KRAS status	Wild-type	40 (51.9)	15 (68.2)	0.268
	Mutant	37 (48.1)	7 (31.8)	
Primary tumour resection	No	7 (8.8)	0 (0.0)	0.336
	Yes	73 (91.2)	22 (100.0)	
Number of intervening treatments*	0-1	34 (42.5)	14 (63.6)	0.129
	≥ 2	46 (57.5)	8 (36.4)	
Third drug concomitant to oxaliplatin retreatment	No	58 (72.5)	15 (68.2)	0.896
	Yes	22 (27.5)	7 (31.8)	
Line of oxaliplatin retreatment	0-2	36 (45.0)	12 (54.5)	0.580
	≥ 3	44 (55.0)	10 (45.5)	
Anti-EGFR concomitant to oxaliplatin retreatment	No	77 (96.2)	19 (86.4)	0.217
	Yes	3 (3.8)	3 (13.6)	
Anti-VEGF concomitant to oxaliplatin retreatment	No	61 (76.2)	18 (81.8)	0.791
	Yes	19 (23.8)	4 (18.2)	
PD while on first oxaliplatin-based regimen	No	72 (90.0)	22 (100.0)	0.272
	Yes	8 (10.0)	0 (0.0)	
First oxaliplatin-based regimen setting	Adjuvant	38 (47.5)	15 (68.2)	0.139
	Metastatic	42 (52.5)	7 (31.8)	

Keys: *= the term “treatments” includes all approaches (medical, surgical or local such as radiotherapy and others).
Legend: PD=progressive disease; SD=stable disease; PR=partial response; CR=complete response.

Supplementary Table S3. Logistic regression analysis of overall disease control rate (DCR) with oxaliplatin retreatment.

Characteristic	Variables	PD (N,%)	SD+PR+CR (N,%)	Significance (p)
Oxaliplatin retreatment disease control rate		43 (42.2)	59 (57.8)	-
Primary tumour location	Right colon	11 (25.6)	20 (33.9)	0.494
	Left colon or rectal	32 (74.4)	39 (66.1)	
Stage at diagnosis	Stage II	3 (7.0)	8 (13.6)	0.475
	Stage III	19 (44.2)	21 (35.6)	
	Stage IV	21 (48.8)	30 (50.8)	
Age at initial diagnosis	Median (IQR)	54.4 (10.8)	56.9 (11.8)	0.279
Gender	Male	22 (51.2)	36 (61.0)	0.430
	Female	21 (48.8)	23 (39.0)	
KRAS status	Wild-type	22 (55.0)	33 (55.9)	1.000
	Mutant	18 (45.0)	26 (44.1)	
Number of intervening treatments*	0-1	12 (27.9)	36 (61.0)	0.002
	≥ 2	31 (72.1)	23 (39.0)	
Third drug concomitant to oxaliplatin retreatment	No	35 (81.4)	38 (64.4)	0.098
	Yes	8 (18.6)	21 (35.6)	
Line of oxaliplatin retreatment	0-2	15 (34.9)	33 (55.9)	0.057
	≥ 3	28 (65.1)	26 (44.1)	
First oxaliplatin-based regimen setting	Adjuvant	22 (51.2)	31 (52.5)	1.000
	Metastatic	21 (48.8)	28 (47.5)	

Keys: *= the term “treatments” includes all approaches (medical, surgical or local such as radiotherapy and others).
Legend: PD=progressive disease; SD=stable disease; PR=partial response; CR=complete response.

Supplementary Table S4. Odds ratios at univariate and multivariate analysis of metastatic colorectal cancer patients who were evaluable for disease control rate (DCR) with oxaliplatin retreatment.

Characteristic	Variables	PD	SD+PR+CR	OR (univariable)	OR (multivariable)
Primary tumour location	Right colon	11 (35.5)	20 (64.5)	-	-
	Left colon	32 (45.1)	39 (54.9)	0.67 (0.27-1.58, p=0.368)	0.61 (0.22-1.62, p=0.329)
Stage at initial diagnosis	Stage II	3 (27.3)	8 (72.7)	-	-
	Stage III	19 (47.5)	21 (52.5)	0.41 (0.08-1.67, p=0.239)	0.28 (0.05-1.38, p=0.135)
	Stage IV	21 (41.2)	30 (58.8)	0.54 (0.11-2.10, p=0.395)	0.67 (0.12-3.15, p=0.626)
Gender	Male	22 (37.9)	36 (62.1)	-	-
	Female	21 (47.7)	23 (52.3)	0.67 (0.30-1.48, p=0.322)	0.63 (0.26-1.50, p=0.302)
KRAS status	Wild-type	22 (40.0)	33 (60.0)	-	-
	Mutant	18 (40.9)	26 (59.1)	0.96 (0.43-2.17, p=0.927)	-
Number of intervening treatments*	0-1	12 (25.0)	36 (75.0)	-	-
	≥ 2	31 (57.4)	23 (42.6)	0.25 (0.10-0.57, p=0.001)	0.27 (0.10-0.66, p=0.005)
Third drug concomitant to oxaliplatin retreatment	No	35 (47.9)	38 (52.1)	-	-
	Yes	8 (27.6)	21 (72.4)	2.42 (0.98-6.46, p=0.064)	2.04 (0.67-6.67, p=0.220)
Line of oxaliplatin retreatment	0-2	15 (31.2)	33 (68.8)	-	-
	≥ 3	28 (51.9)	26 (48.1)	0.42 (0.18-0.94, p=0.037)	-
First oxaliplatin-based regimen setting	Adjuvant	22 (41.5)	31 (58.5)	-	-
	Metastatic	21 (42.9)	28 (57.1)	0.95 (0.43-2.08, p=0.890)	0.75 (0.26-2.13, p=0.596)

Supplementary Figure S1. Forest plot depicting the hazard ratios of the multivariate analysis of oxaliplatin retreatment disease control rate (DCR).

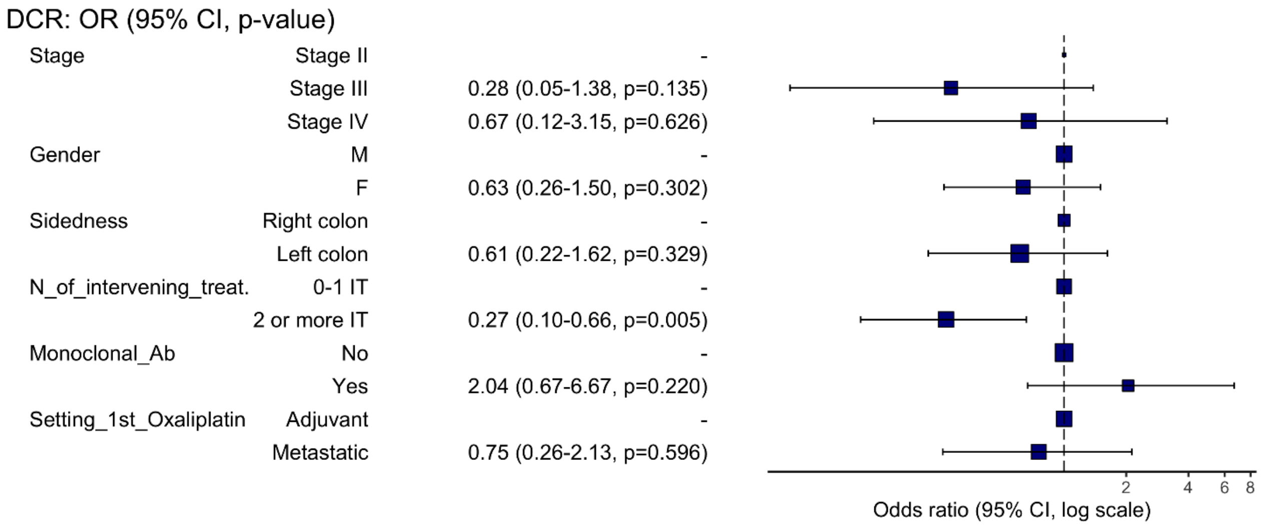

Supplementary Figure S2. Prevalence of standard (*RAS*, *BRAF* and *MMR*) and DNA damage response (DDR) alterations in 6 metastatic colorectal cancer (mCRC) patients who underwent next generation sequencing (NGS).

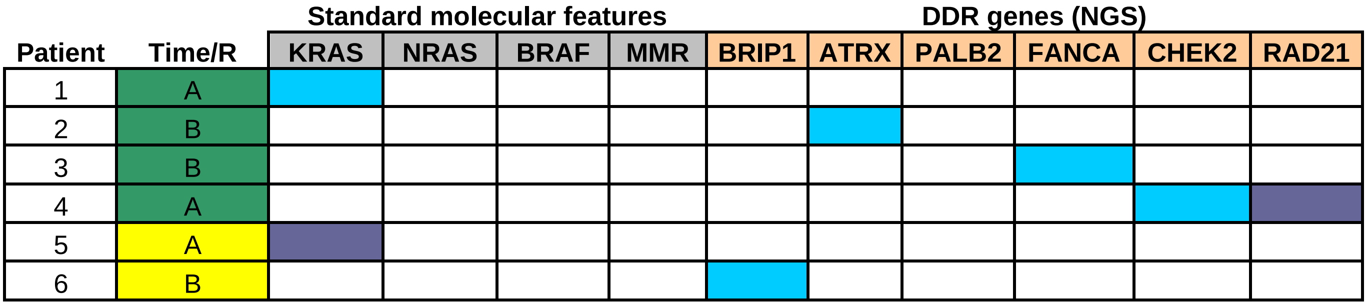

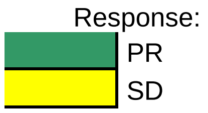

Legend: Timing=timing of tumor tissue sampling compared to oxaliplatin retreatment. R=response; A=after oxaliplatin retreatment; B=before oxaliplatin retreatment; Blue dot=mutant; Violet dot=amplified. List of DNA damage response (DDR) genes included in the panel of Foundation Medicine Assay next generation sequencing (NGS): ARID1A, ATM, ATR, ATRX, BAP1, BARD1, BRCA1, BRCA2, BRIP1, CHEK1, CHEK2, FANCA, FANC, FANCG, FANCL, PALB2, PARP1, PARP2, PARP3, PPARG, PPP2R1A, PPP2R2A, RAD21, RAD51, RAD51B, RAD51C, RAD51D, RAD52, RAD54L, TIPARP, XRCC2
